# Towards sustainable diagnostics: replacing unstable H_2_O_2_ by photoactive TiO_2_ in testing systems for visible and tangible diagnostics for use by blind people

**DOI:** 10.1039/c8ra06711b

**Published:** 2018-11-09

**Authors:** Yulia V. Lanchuk, Sviatlana A. Ulasevich, Tatiana A. Fedotova, Dmitry M. Kolpashchikov, Ekaterina V. Skorb

**Affiliations:** ITMO University Lomonosova St. 9 191002 St. Petersburg Russian Federation skorb@corp.ifmo.ru; Chemistry Department University of Central Florida Orlando FL USA

## Abstract

Blind and color blind people cannot use colorimetric diagnostics; the problem is especially severe in rural areas where high temperatures and the absence of electricity challenge modern diagnostics. Here we propose to replace the unstable component of a diagnostic test, H_2_O_2_, with stable TiO_2_. Under UV irradiation, TiO_2_ forms reactive oxygen species that initiate polymerization of acrylamide causing liquid-to-gel transition in an analyte-dependent manner. We demonstrate that specific DNA sequences can be detected using this approach. This development may enable the detection of biological molecules by users with limited resources, for example in developing countries or for travelers in remote areas.

## Abstract 2

Blind and color blind people cannot afford colorimetric diagnostics; the problem is especially severe in rural areas where high temperatures and the absence of electricity challenge modern diagnostics.

## Graphical abstract

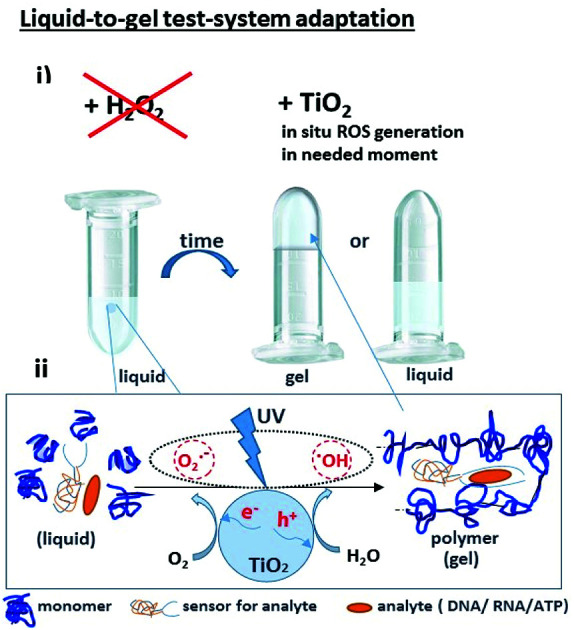

An ideal diagnosis, including diagnosis for infectious diseases, should meet the ASSURED criteria: (i) affordable by those at risk of infection; (ii) sensitive; (iii) specific; (iv) user-friendly; (v) rapid, and robust, for example not requiring refrigerated storage; (vi) equipment-free; (vii) delivered to those who need it.^1^

Analytic methods with visually detectable outputs (*e.g.* color change) satisfy criteria (iv) and (vi), and are therefore among the most common. Indeed, pregnancy tests, test strips for measuring acetone and glucose in urine for diabetic people, and pH strips are known to be the best to make the analysis easiest in data output. However, such methods cannot be used by the visually impaired.

Recently, we described an alternative output signal that cannot only be detected by sight, but also by touch and applied it in the detection of adenosine triphosphate (ATP) and deoxyribonucleic acid (DNA).^2^ The method is based on the analyte-dependent radical polymerization of acrylamide into polyacrylamide in the presence of hydrogen peroxide (H_2_O_2_). H_2_O_2_ serves as a source of radicals. The test uses affordable reagents and does not require any instrumentation for signal readout. Such test systems can be adopted for detection of a wide variety of biological analytes. Unfortunately, H_2_O_2_ is subject to light decomposition and should be refrigerated for long term storage; it is also prone to exploding at high concentrations. Therefore, substituting H_2_O_2_ with a more stable ingredient would increase the shelf life of the test system and make it usable in those environments with limited access to refrigeration. This work is devoted to addressing the ASSURED criterion (v): we substituted perishable H_2_O_2_ with stable titania (TiO_2_) as an alternative source of initiators of radical polymerization.

Recently we demonstrated the possibility of converting electromagnetic energy into pH gradients^3^ or generating reactive oxygen species (ROS) using TiO_2_,^3,4^ which makes this approach attractive for controlling interactions between chemical networks. Moreover, combining several functional chemical networks can result in a network with new functions. We have also shown that the pH gradient on titania can be used for regulating enzymatic reaction networks.^5^ In this work, we were interested in using photogenerated ROS (such as superoxide anion O_2_˙^−^, hydroxyl radical ˙OH, and hydrogen peroxide H_2_O_2_) (Fig. 1) to initiate radical polymerization with the aim of making a visual and tactile portable sensor for the detection of biologically important molecules (DNA, RNA, ATP). Under ultraviolet (UV) irradiation, TiO_2_ splits water, which results in the generation of a high concentration of ROS and free radicals.^6^

**Fig. 1:**
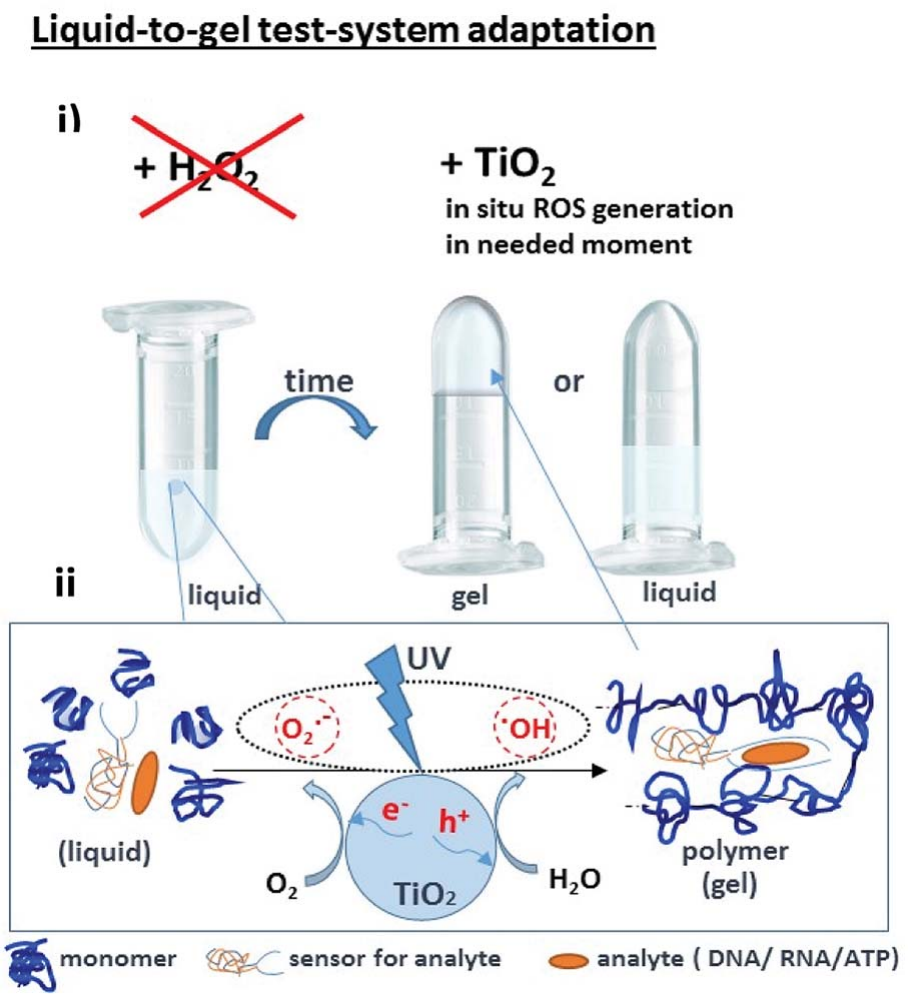
(i) A portable sensor based on a light-induced liquid-to-gel transition for polymer by radical polymerization on TiO_2_ particles. The details of the sensor design are shown in Fig. 5; (ii) ROS formation on titania *via* reactions with photogenerated photohole (h^+^) and photoelectron (e^−^).

The sensing system is based on deoxyribozyme sensor that produces ROS in the presence of a specific analyte, thereby triggering the radical polymerization of acrylamide into polyacrylamide. The buffer contained acetylacetone, H_2_O_2_, 40% acrylamide/bisacrylamide, hemin and a split deoxyribozyme sensor with peroxidase-like activity (PxR).^2^ In the presence of the specific analyte sequence (A1 in this study), the sensor hybridized with the analyte and formed PxR, which bound hemin and decomposed H_2_O_2_ to ROS. The latter oxidized acetylacetone to the acetylacetone radical, which initiated the polymerization of acrylamide, resulting in liquid-to-gel conversion. Here, the aim of the research is to increase the system sustainability by changing H_2_O_2_ to TiO_2_ particles. Noteworthy is that the system is externally controlled by UV light, since TiO_2_ produces ROS only upon irradiation.

Firstly, we quantified the TiO_2_-derived ROS with the aim of finding the optimal conditions for the generation of the minimum amount of ROS needed for the polymerization. We used luminol chemiluminescence (CL) calibrated with hydrogen peroxide. Luminol (5-amino-2,3-dihydro-1,4-phthalazinedione) is a widely used CL reagent and has CL emission at different wavelengths depending on the conditions.^6^ Traditionally, luminol CL is observed in the presence of H_2_O_2_ in alkaline solutions (Fig. 2), which is catalyzed by metal ions, metal complexes or vitamins.^7–10^

**Fig. 2:**
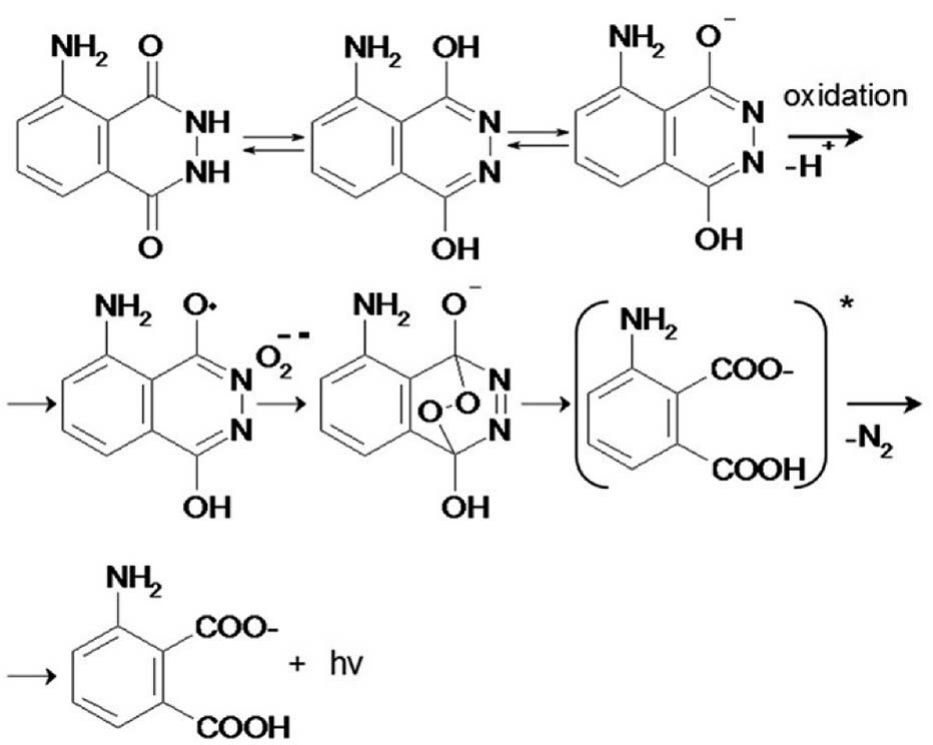
Mechanism of chemiluminescence of luminol and O_2_^−^˙.

The luminol CL signaling is conveniently used for the detection of ROS (particularly superoxide anion, hydrogen peroxide, hydroxyl radical) in biological systems.^11–16^

Irradiation of TiO_2_ suspensions of different concentrations (0.03 M and 0.06 M) at two wavelengths (365 nm and 254 nm) revealed more ROS being formed upon irradiation at 254 nm – as was expected (Fig. 3).

**Fig. 3:**
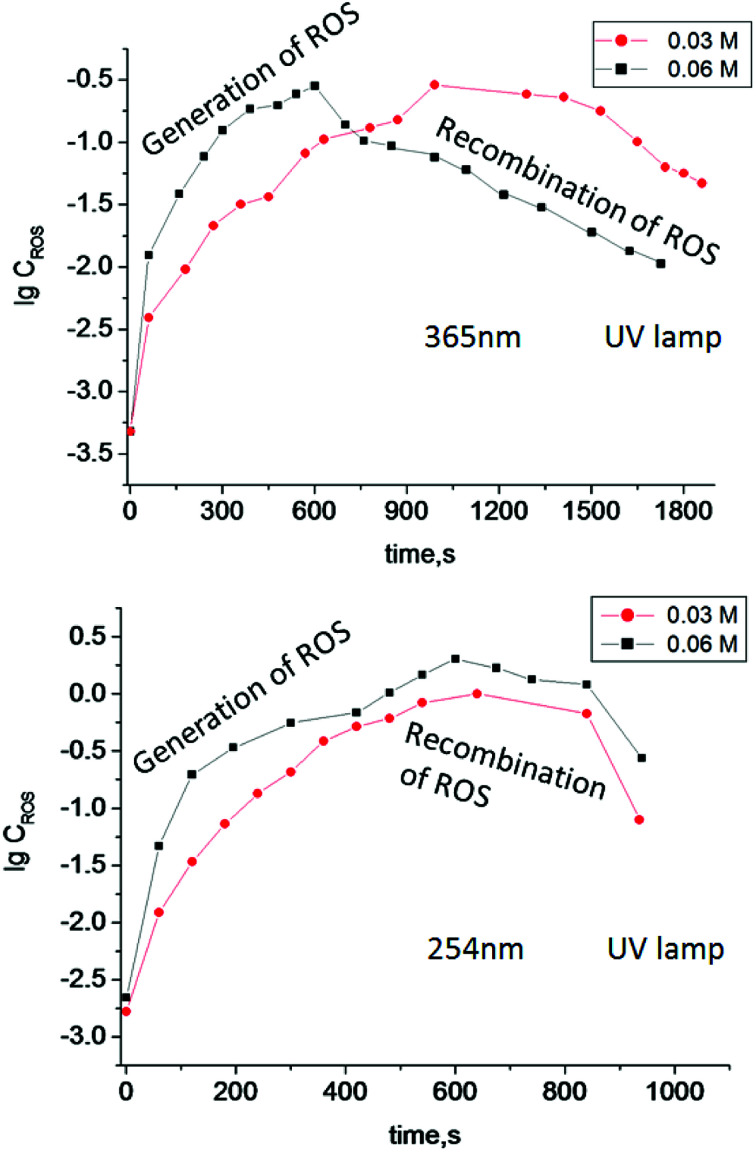
Log concentration of ROS generated in TiO_2_ suspensions of different concentration (0.03 M and 0.06 M) under irradiation by UV light with different wavelengths *vs.* irradiation time.

According to the data obtained, the minimum concentration of TiO_2_ needed to trigger polymerization after 5 min of irradiation at 365 nm was 1.25 mM (Fig. 4). We therefore used these conditions in the following experiments, since short irradiation time and longer wavelength of UV-A irradiation are less damaging to the DNA-based biosensor component than UV-C light with wavelength of 254 nm. It should be noted that control experiments were made without TiO_2_ particles and polymerization did not occur. Next, we optimized the conditions for the analyte-dependent activation of PxR resulting in acrylamide polymerization. If polymerization occurs, the gel will stick to the bottom of the inverted tubes (Fig. 5).^2^ To demonstrate the general applicability of the approach, we designed a sensor for an analyte of biomedical significance, nucleic acids, as an example. A sequence of 16S rRNA, which was represented in this study by synthetic A1 sequence (5′-CAT TAC TCA CCC GTC CGC CAC TCG TCA GCG AAG CAG CAA GCT GCT TCC TGT TAC CGT TCG), of pathogenic *E. coli* O157:H7 was chosen as the target analyte,. The binding of A1 to PxR1 and PxR2 stabilized the G-quadruplex structure, which then binds to hemin and catalyzes the polymerization (Fig. 5A).

**Fig. 4:**
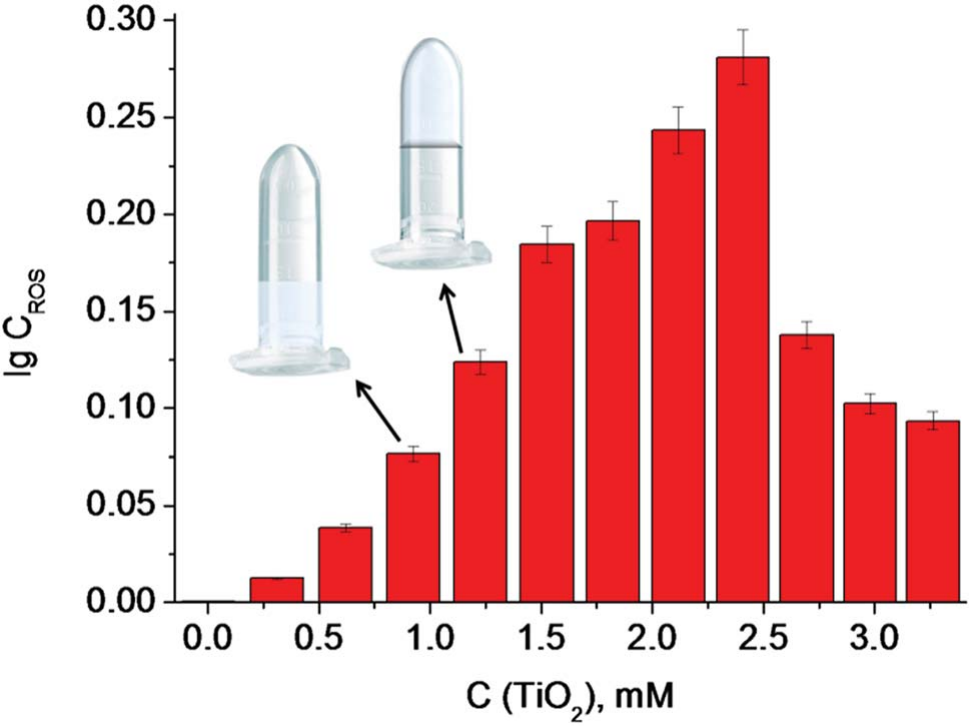
The dependence of the log concentration of ROS generated on TiO_2_ particles on TiO_2_ suspension concentration. Inserts show the minimum concentration of TiO_2_ particles in a suspension (therefore minimum concentration of ROS generated on TiO_2_ particles), needed for the gel formation.

**Fig. 5:**
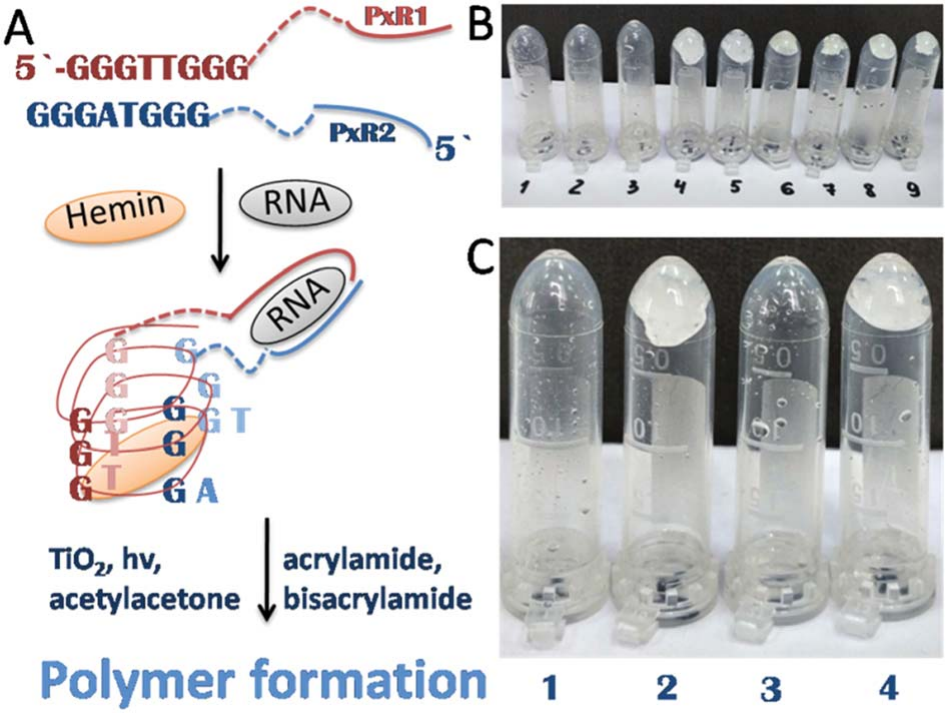
Polymerization-based visual and tactile detection of A1 analyte. (A) Sensor design: RNA strands PxR1 and PxR2 bind A1 sequence and form a G-quadruplex structure, which then binds to hemin (green oval) and catalyzes the radical polymerization of the acrylamide solution. The dotted lines represent triethylene glycol linkers. (B and C) Inverted test-tubes with radical polymerization of acrylamide initiated by reactive oxygen species generated on TiO_2_ particles under UV light 365 nm. (B) Systems with different concentrations of hemin (Table 1). (C) All samples contained hemin of 5 × 10^−8^ M (1); (2) with G-quadruplex; (3) with RNA strands PxR1 (1 μM) and PxR2 (1 μM); (4) with PxR1 and PxR2 and RNA (1 μM).

Firstly, we tested the influence of hemin concentration on polymer formation. Gel fragments were observed in the presence of hemin at the concentration higher than 5 × 10^−8^ M (Table 1). No gel fragments were observed in the absence of TiO_2_ or hemin (two control experiments). Gel formation was observed in the system with the buffer components, hemin and TiO_2_.

**Table 1:** Influence of hemin concentration on polymer formation

No.	1	2	3	4	5	6
*C*, M	10^−9^	2.5 × 10^−8^	5 × 10^−8^	7.5 × 10^−8^	10^−7^	2.5 × 10^−7^
Gel	−	−	−	+	+	+

Sample 1 (negative control) with a hemin concentration of 5 × 10^−8^ M shows no gel formation (Fig. 5c), sample 2 (positive control) contained already formed G-quadruplex which triggered the polymerization reaction. Sample 3 contained separated RNA fragments of PxR1 and PxR2 without A1 analyte: no gel formation was observed. The presence of A1 analyte in sample 4 resulted in polymerization. Therefore, we achieved analyte-dependent polymer formation without using H_2_O_2_. This development is pivotal for the future development of H_2_O_2_-free tests systems for nucleic acid analysis employable by blind and color blind people.

Reactions on the surface are very attractive when considering the development of a robust sensor. The surface layer of anodized titania nanotubes (TNT) is suitable for surface ROS generation.^17^ To understand how much ROS were generated on TNT surfaces, experiments with luminol CL were conducted (Fig. 6). A benefit of using light is the ability to control reaction networks with an external stimulus. To check our hypothesis of using TNT as a good alternative source of ROS and understand how much photogenerated ROS are needed for radical polymerization, we experimented with the buffer solution (Fig. 6B) (neither analyte nor sensor for the analyte were present). Polymerization was completed after 15 min of UV irradiation (365 nm), which proves the use of TNT as an alternative robust surface for ROS generation. This polymerized gel can also find its application as protective coating against unfavorable environment.

**Fig. 6:**
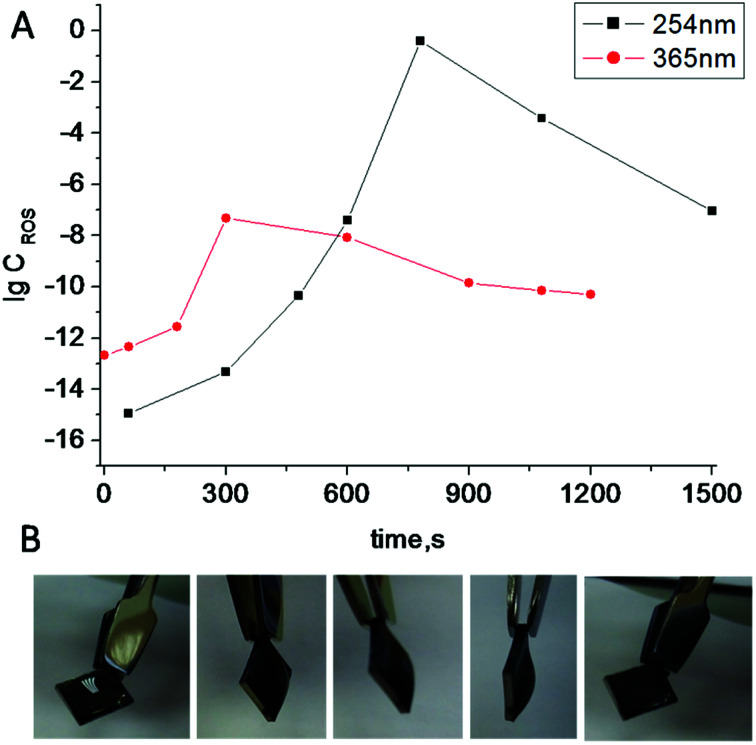
(A) Log concentration of ROS generated on TiO_2_ nanotubes (TNT) under UV irradiation at different wavelengths *vs.* time. (B) Radical polymerization of acrylamide initiated by ROS generated on TNT under UV irradiation at 365 nm.

In order to demonstrate this hypothesis, a photolithography displaying the name of our laboratory was made (Fig. 7).

**Fig. 7:**
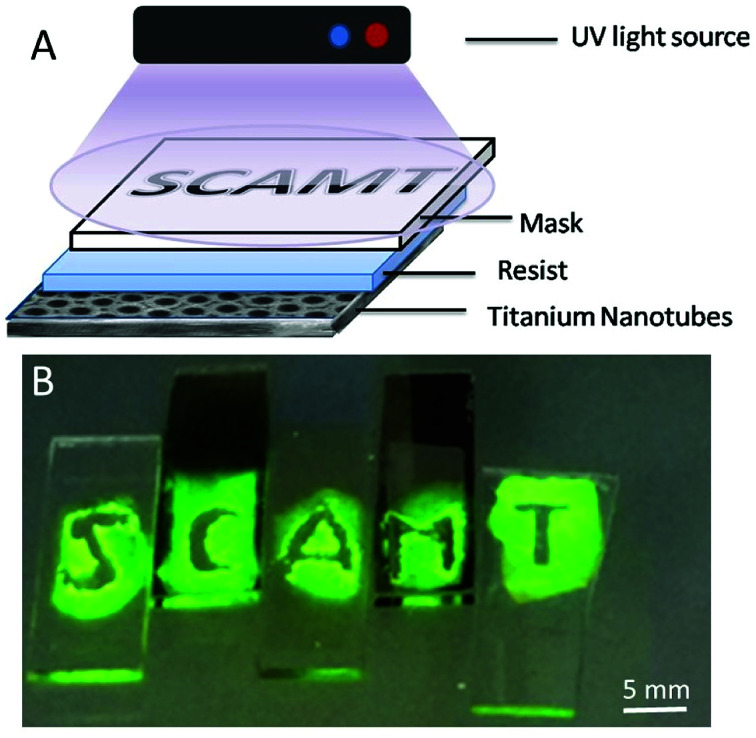
(A) Scheme of photolithography process (negative resist) by radical polymerization of acrylamide initiated by ROS generated on TiO_2_ anodized surface (TNT) under UV light 365 nm; (B) experiment of polymerization of test-sample on TNT allows further test improvement.

Nowadays, methods for the detection of biological analytes are under continuous development.^18,19^ Sensors usually display their readings on a screen or by some visual means. This interface is not user-friendly for the visually impaired. Here, we report improvement on an alternative method that can be used for the tactile detection of biological analytes and, therefore, is more user-friendly for blind people. Using this method, we can detect nucleic acids as well as analytes that are used in the diagnosis of infectious diseases. The use of PxR-based (or PxD, *etc.*) sensors are widely spread in the detection of a wide variety of analytes, including small molecules, metal ions and proteins.^20^

It should be noted that all the analytes can be detected with high specificity, even at room temperature, which is important in practice. The main disadvantage of this assay is its sensitivity to atmospheric oxygen, which inhibits polymerization^16^ and makes the analysis difficult. We hope that future progress in creating special equipment for carrying out such experiments in an oxygen-free environment may help to solve this problem.

The toxicity of liquid acrylamide can be easily circumvented by wearing gloves. We also consider that the reported assay in this article is very promising beyond tactile-like sensors. For example, the liquid-to-gel transition can be used with light in the regulation of liquid flow in the channels of (micro)fluidic devices.

In conclusion, point-of-care diagnostic systems should be cost efficient, easy to transport, robust and sustainable among other characteristics. To improve these qualities, we replaced unstable hydrogen peroxide component with stable and robust titania in the liquid-to-gel testing system. The ability to trigger the system by light irradiation is an attractive alternative to traditional tests in which the reaction is triggered by the addition of analytes. We hope that the method developed in this study will make home test-systems available to blind and color blind persons.

## Experimental section

### Materials

Hombikat UV100 (>99% anatase, 5 nm particle size, supplied by Sachtleben Chemie); luminol, hemin, DMSO (99.9%, anhydrous (max. 0.005% H_2_O)) and sodium hydroxide (NaOH) were acquired from Sigma Aldrich. Millipore water (Milli-Q Plus 185) was used for preparation of aqueous solutions and sample washing. RNAse-free water was purchased from Fisher Scientific, Inc. (Pittsburgh, PA) and used for all buffers and for the stock solutions of oligonucleotides. The oligonucleotides were obtained from Integrated DNA Technologies, Inc. (Coralville, IA). The oligonucleotides were dissolved in water and stored at −20 °C until needed. Stock concentrations of oligonucleotides were calculated by measuring the absorption of the solutions at 260 nm by using a Perkin-Elmer Lambda 35 UV/Vis spectrometer (San Jose, CA). Extinction coefficients of oligonucleotides were calculated by using OligoAnalyzer 3.1 software (Integrated DNA Technologies, Inc.). All experiments were carried out in the buffer, containing 50 mM HEPES pH 6.6., 50 mM MgCl_2_, 20 mM K^+^, 120 mM NaCl, 1% DMSO v/v, 0.03% w/v Triton X 100, 38% w/v acrylamide, 2% w/v bisacrylamide, 127 mM acetylacetone.

### Chemiluminescence measurements

3 mL of TiO_2_ suspension (0.03 M and) or a drop of pure water (100 μL) on TNT was irradiated with the UV lamp (with different wavelength 365 nm and 254 nm) and every minute a 10 μL aliquot of the irradiated solution was taken and added to a cuvette with 2 mL of 0.2% luminol in 1% NaOH solution for CL measurements. The fluorescence spectra were measured from 380 to 550 nm at the excitation wavelength of 310 nm. The widths of emission and excitation slits were set at 10.0 and 10.0. The fluorescence spectra were measured by a Cary Eclipse fluorescence spectrophotometer equipped with a 1.0 cm quartz cell.

### Polymerization in tubes

1 mL liquid in a tube contained 50 mM HEPES pH 6.6, 50 mM MgCl_2_, 20 mM K^+^, 120 mM NaCl, 1% DMSO v/v, 0.03% w/v Triton X 100, 38% w/v acrylamide, 2% w/v bisacrylamide, 127 mM acetylacetone and 5 × 10^−8^ M hemin. 3 mL of TiO_2_ suspension was irradiated under UV light at 365 nm during 15 min and then added to the tube with all the components. The final concentration of TiO_2_ in tube was 1.25 mM. Tactile readout is possible if the samples are placed on a piece of filter paper. The experiment was carried out in box with argon due to the fact that oxygen inhibits the polymerization reaction.^16^

### Polymerization on the surface

The drop on the TNT contained reaction buffer: 50 mM HEPES pH 6.6, 50 mM MgCl_2_, 20 mM K^+^, 120 mM NaCl, 1% DMSO v/v, 0.03% w/v Triton X 100, 38% w/v acrylamide, 2% w/v bisacrylamide, 127 mM acetylacetone, 5 × 10^−8^ M hemin. Experiment was carried out in box with argon due to the fact that oxygen inhibits the polymerization reaction. Radical polymerization of acrylamide was initiated by ROS generated on TNT under UV irradiation at 365 nm during 15 min. Photolithography was made the same way with photomasks.

## Conflicts of interest

There are no conflicts to declare.

## Supplementary Material
